# ﻿Unveiling species diversity within early-diverging fungi from China IV: Four new species of *Absidia* (Cunninghamellaceae, Mucoromycota)

**DOI:** 10.3897/mycokeys.119.147816

**Published:** 2025-06-25

**Authors:** Zi-Ying Ding, Xin-Yu Ji, Meng-Fei Tao, Yang Jiang, Wen-Xiu Liu, Yi-Xin Wang, Zhe Meng, Xiao-Yong Liu

**Affiliations:** 1 College of Life Sciences, Shandong Normal University, Jinan, 250358, China Shandong Normal University JInan China; 2 State Key Laboratory of Mycology, Institute of Microbiology, Chinese Academy of Sciences, Beijing 100101, China Chinese Academy of Sciences Beijing China

**Keywords:** Basal fungi, fungal diversity, molecular phylogeny, Mucorales, taxonomy

## Abstract

To investigate early-diverging fungi in soil from Yunnan Province, China, four novel species of the genus *Absidia* were discovered based on a combination of morphological characteristics, molecular evidence, and physiological features. Molecular phylogenetic analyses of SSU–ITS–LSU–Act–TEF1α sequences indicate that *Absidiaarrhiza***sp. nov.**, *A.simplex***sp. nov.**, *A.sphaerica***sp. nov.**, and *A.viridis***sp. nov.** are closely related to *A.chinensis*, *A.panacisoli*, *A.medulla*, and *A.varians*, respectively. *A.arrhiza* is named for the sparse presence of rhizoids. *A.simplex* refers to the simple branching of its sporangiophores. *A.sphaerica* is characterized by its spherical columellae. *A.viridis* is distinctive for its light green colony. These four new species are described and illustrated herein, and their phenotypic and genotypic differences from allied species are discussed.

## ﻿Introduction

The genus *Absidia* Tiegh. (Cunninghamellaceae, Mucorales) was proposed by van Tieghem (1876) and typified with *A.reflexa* Tiegh. *Absidia* species are distributed worldwide in soils, herbivorous animal feces, decaying material, and air (Zhang et al. 2018; Cordeiro et al. 2020; Lima et al. 2020; Hurdeal et al. 2021; Zhao et al. 2021, 2023). Some species of *Absidia* are capable of producing various secondary metabolic substances for industrial and medicinal applications, such as fatty acids, α-galactosidase, chitin, and chitosan (Kaczmarek et al. 2019; Chen et al. 2020).

The genus *Absidia* possesses remarkable morphological features, mainly in the sporangia, sporangiophores, rhizoids, and stolons. Sporangiophores generally arise from stolons and are erect or curved, unbranched, simply branched, sympodially branched, or arranged in whorls. Sporangia are multispored, deliquescent-walled, pyriform to globose, and located at the apex of the sporangiophores. Columellae bear one to two projections at their tips. Zygospores are formed through mutual attraction and the development and fusion of two gametangia (Hoffmann et al. 2007; Zhao et al. 2023).

The circumscription of the genus *Absidia* has undergone continuous revision over the past century. It was originally classified in Absidiaceae (von Arx 1982), then transferred to Mucoraceae (Benny et al. 2001), and later placed in Cunninghamellaceae (Ashton 2009). *Absidia* sensu lato is now divided into three genera: *Absidia* sensu stricto, *Lentamyces*, and *Lichtheimia* (Hoffmann et al. 2007; Hoffmann and Voigt 2009; Hoffmann 2010). To date, 142 names have been recorded in *Absidia* globally, especially in Australia, Argentina, Brazil, China, Estonia, Lithuania, Korea, and Czechia (http://www.indexfungorum.org/, accessed on 30 October 2024). In China, Zong et al. (2021a, b), Zhao et al. (2021, 2022a, b, 2023), Tao et al. (2024), Luo et al. (2024), and Zhang et al. (2018) reported 13, 22, three, one, and one new *Absidia* species, respectively. These reports have greatly enriched the known species diversity of *Absidia* in China.

In this study, four novel species – *Absidiaarrhiza* sp. nov., *A.simplex* sp. nov., *A.viridis* sp. nov., and *A.sphaerica* sp. nov. – are described based on morphological characteristics, molecular evidence, and physiological features. This represents the fourth report in a series on the diversity of early-diverging fungi from China (Tao et al. 2024; Wang et al. 2024; Zhao et al. 2024).

## ﻿Materials and methods

### ﻿Sample collection and strain isolation

Soil samples were collected from Yunnan Province in China in July 2024, following the methods of Li et al. (2020) and Zou et al. (2022). Approximately 50 g of soil was kept in a sterile plastic bag, labeled with number, date, vegetation type, altitude, latitude, and longitude. All samples were preserved at 4 °C upon delivery to the laboratory. These samples were isolated and purified by soil dilution plate and single spore isolation methods (Zhao et al. 2022a). In brief, 10 g soil samples were transferred into a conical bottle containing 90 mL sterile water and mixed with a shaker at 20 °C and 120 rpm for 20 minutes, resulting in a soil suspension with a dilution ratio of 10^−1^. A pipette gun was adopted to transfer 1 mL soil suspension into a test tube containing 9 mL sterile water to yield 10^−2^ soil suspensions. The aforementioned steps were repeated to obtain 10^−3^ and 10^−4^ soil suspensions. A 200 µL volume of soil suspension at a concentration of 10^−4^ was pipetted onto the center of Rose Bengal Chloramphenicol agar (RBC: peptone 5.00 g/L, glucose 10.00 g/L, MgSO4·7H2O 0.50 g/L, KH2PO4 1.00 g/L, rose bengal 0.05 g/L, chloramphenicol 0.10 g/L, and agar 15.00 g/L; Corry et al. 2003), evenly dispersed with a sterile glass triangle coater, and cultivated in a dark incubator at 25 °C for 3–5 days. Subsequently, an agar portion with fungal mycelia at the frontier of the colony was inoculated on a new potato dextrose agar plate (PDA: 200 g potato, 20 g dextrose, 20 g agar, 1000 mL distilled water, pH 7.0) and cultured at 25 °C in a dark environment. All strains were stored at 4 °C in 10% sterilized glycerol tubes. The dried cultures of holotypes were deposited in the Herbarium Mycologicum Academiae Sinicae, Beijing, China (**HMAS**). The living cultures of ex-holotypes were deposited in the China General Microbiological Culture Collection Center, Beijing, China (**CGMCC**). At the same time, all strains involved were preserved in the Shandong Normal University, Jinan, China (**XG**).

### ﻿Morphology and maximum growth temperature

Microscopic morphological characteristics of fungi were observed by a stereomicroscope (Olympus SZX10, OLYMPUS, Tokyo, Japan) and an optical microscope (BX53, Olympus, Tokyo, Japan). The structure of fungi was photographed with a high-definition color digital camera (DP80, Olympus, Tokyo, Japan). Morphological characteristics were described using more than 20 measurements. The maximum growth temperature tests were performed following the methods of predecessors (Zheng et al. 2007, 2009a; Zheng and Liu 2009b; Zong et al. 2021a; Zhao et al. 2022b). To determine the maximum growth temperature for each strain, three plates were incubated for two days at 25 °C and then incubated at an increased temperature gradient of 1 °C until the colonies did not grow further. The taxonomic information of the target strains was uploaded to the Fungal Name repository (https://nmdc.cn/fungalnames/).

### ﻿DNA extraction, PCR amplification, and sequencing

Fungal genomic DNA was extracted by the CTAB (cetyl trimethyl ammonium bromide) method and the Beaver Beads Plant DNA Kit (Cat. No.: 70409-20; Beaver Biomedical Engineering Co., Ltd.; Doyle and Doyle 1990; Guo et al. 2000). The ITS rDNA (internal transcribed spacer of ribosomal DNA), LSU rDNA (large subunit of ribosomal DNA), Act (actin), TEF1α (translation elongation factor 1 alpha), and SSU rDNA (small subunit of ribosomal DNA) were amplified with the primer pairs under the procedure of polymerase chain reaction (PCR) in Table 1. The amplification reaction was carried out in a 25 µL reaction system containing 12.5 µL 2× Hieff Canace® Plus PCR Master Mix (Yeasen Biotechnology, Cat. No. 10154ES03, Shanghai, China), 1 µL each for forward and reverse primers (10 µM) (TsingKe, Qingdao, China), 1 µL template genomic DNA (about 1 µM), and 9.5 µL ddH2O. Then, the PCR-amplified products were analyzed by 1% agarose gel electrophoresis with a Gel Extraction Kit (Cat# AE0101-C; Shandong Sparkjade Biotechnology Co., Ltd., Jinan, China) and observed under ultraviolet light (Zhang et al. 2022). Sanger sequencing was performed by Tsingke Biotechnology Co., Ltd. (Qingdao, China).

**Table 1.** PCR primers and program used in this study.

Loci	PCR primers	Primer sequence (5’ – 3’)	PCR cycles	References
SSU	NS1	GTA GTC ATA TGC TTG TCT CC	(95 °C: 60 s, 54 °C: 50 s, 72 °C: 1 min) × 35 cycles	(White et al. 1990)
	NS4	CTT CCG TCA ATT CCT TTA AG		
ITS	ITS5	GGA AGT AAA AGT CGT AAC AAG G	(95 °C: 30 s, 55 °C: 30 s, 72 °C: 1 min) × 35 cycles	(White et al. 1990)
	ITS4	TCC TCC GCT TAT TGA TAT GC		
LSU	LR0R	GTA CCC GCT GAA CTT AAG C	(95 °C: 30 s, 51 °C: 30 s, 72 °C: 1 min) × 35 cycles	(Hurdeal et al. 2023)
	LR5	TCC TGA GGG AAA CTT CG		
*Act*	Act-1	TGG GAC GAT ATG GAI AAI ATC TGG CA	(95 °C: 30 s, 52 °C: 30 s, 72 °C: 1 min) × 35 cycles	(Carbone and Kohn. 1999)
	Act-4R	TCI TCG TAT ICT IGC TII GAI ATC CAC AT		
*TEF-1α*	EF1-983F	GCY CCY GGH CAY CGT GAY TTY AT	(95 °C: 30 s, 48 °C: 30 s, 72 °C: 1 min) × 35 cycles	(Jaklitsch et al. 2005)
	TEF1LLErev	AAC TTG CAG GCA ATG TGG		

### ﻿Phylogenetic analyses

The obtained DNA sequences were assembled and proofread with the software MEGA v.7.0 (Kumar et al. 2016), and the GenBank accession numbers for all sequences used in this article were recorded in Suppl. material 1: table S1. All sequences were then submitted to the NCBI GenBank nucleotide database for similarity comparison. Reference sequences were retrieved from GenBank according to newly published articles (Tao et al. 2024). Phylogenetic analyses of *Absidia* were performed using maximum likelihood (ML), maximum parsimony (MP), and Bayesian inference (BI) methods. MrModelTest v2.3 was used to select the optimal evolutionary models for each locus (Nylander 2004), which were then implemented in partitioned BI analyses. The ML analysis was performed on the CIPRES Science Gateway website (https://www.phylo.org/) using RAxML-HPC2 on XSEDE v.8.2.4 (Stamatakis 2014) with 1,000 bootstrap replicates. MP analysis was executed using default parameters (Swofford 2002). BI analysis was performed on a Linux system server, utilizing a quick start configuration with an automatic stop option (Huelsenbeck and Ronquist 2001; Nylander 2004). Bayesian inference was implemented with four parallel runs, each consisting of 5,000,000 generations with a sampling frequency of once every 100 generations. The burn-in score of 0.25 was set, and posterior probabilities (PP) were calculated from the remaining trees. The phylogenetic trees were uploaded to the iTOL website (https://itol.embl.de) for layout and then submitted to Adobe Illustrator CC 2019 for beautification.

## ﻿Results

### ﻿Phylogeny

**Figure 1.**
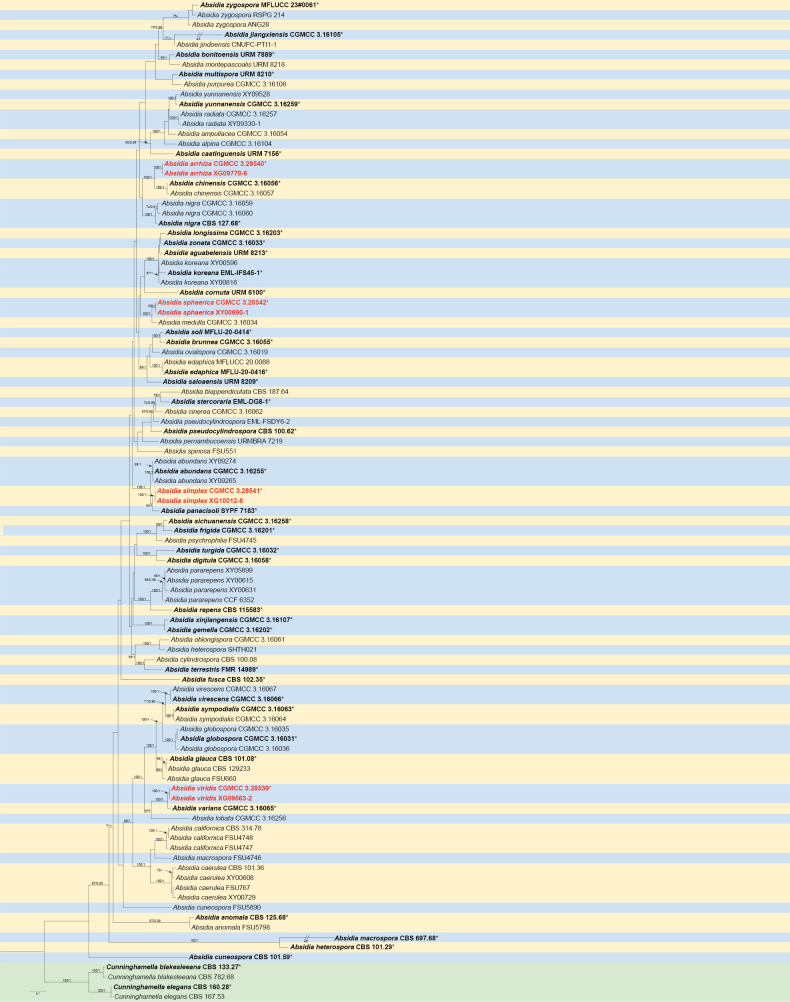
The maximum likelihood phylogenetic tree of *Absidia* based on concatenated sequences of ITS rDNA, LSU rDNA, TEF1α, Act, and SSU rDNA, with *Cunninghamellablakesleeana* and *C.elegans* as outgroups. Maximum likelihood bootstrap values (MLBV ≥ 70%) and Bayesian inference posterior probabilities (BIPP ≥ 0.9) are shown at the nodes, separated by slashes “/”. Ex-type or ex-holotype strains are indicated in bold black with an asterisk “*”. Strains obtained in this study are highlighted in red. Some branches are shortened for overall tree layout, indicated by “//”, with “×” denoting the fold of shortening. The scale bar at the bottom left represents substitutions per site.

The molecular dataset comprised a total of 101 strains, including 36 *Absidia* species and two outgroup species of *Cunninghamella*: *C.blakesleeana* (CBS 782.68) and *C.elegans* (CBS 167.53). The concatenated alignment contained 4,909 characters, consisting of ITS rDNA (positions 1–964), LSU rDNA (965–1,975), TEF1α (1,976–2,980), Act (2,981–3,887), and SSU rDNA (3,888–4,909). Among these, 2,680 characters were constant, 701 were variable but parsimony-uninformative, and the remaining 1,528 were parsimony-informative. Bayesian inference was performed using the GTR + I + G model. The maximum likelihood (ML) tree topology was highly congruent with the Bayesian inference (BI) tree topology. The ML tree is presented here as a representative (Fig. 1), and the ML tree based solely on the ITS rDNA sequences of *Absidia* is provided in Suppl. material 1: fig. S1. Eight *Absidia* strains isolated in this study formed four distinct, independent clades. *A.arrhiza* was closely related to *A.chinensis* (MLBV = 100, BIPP = 1.00). *A.simplex* clustered closely with *A.panacisoli* (MLBV = 100, BIPP = 1.00). *A.viridis* was sister to *A.varians* (MLBV = 100, BIPP = 1.00). Finally, *A.sphaerica* formed a sister clade to *A.medulla* (MLBV = 100, BIPP = 1.00).

### ﻿Taxonomy

The morphological characteristics, molecular sequences, and physiological features of four new species of *Absidia* were described as follows.

#### ﻿ Absidia arrhiza

Taxon classificationFungiMucorales

Z.Y. Ding, Yang Jiang, Yi Xin Wang & X.Y. Liu sp. nov.

79183AF7-7CB8-5EE0-AADE-A2475353FAE9

Fungal Names: FN 572259

Fig. 2

##### Type.

China • Yunnan Province, Xishuangbanna Dai Autonomous Prefecture, Menghai County G219 (Xijing Line) (22°11'02"N, 100°17'22"E, altitude 1492.96 m), from soil, 7 July 2024, Z.Y. Ding and X.Y. Liu, holotype HMAS 353363, ex-holotype living culture CGMCC 3.28540 (= XG09770-7).

**Figure 2.**
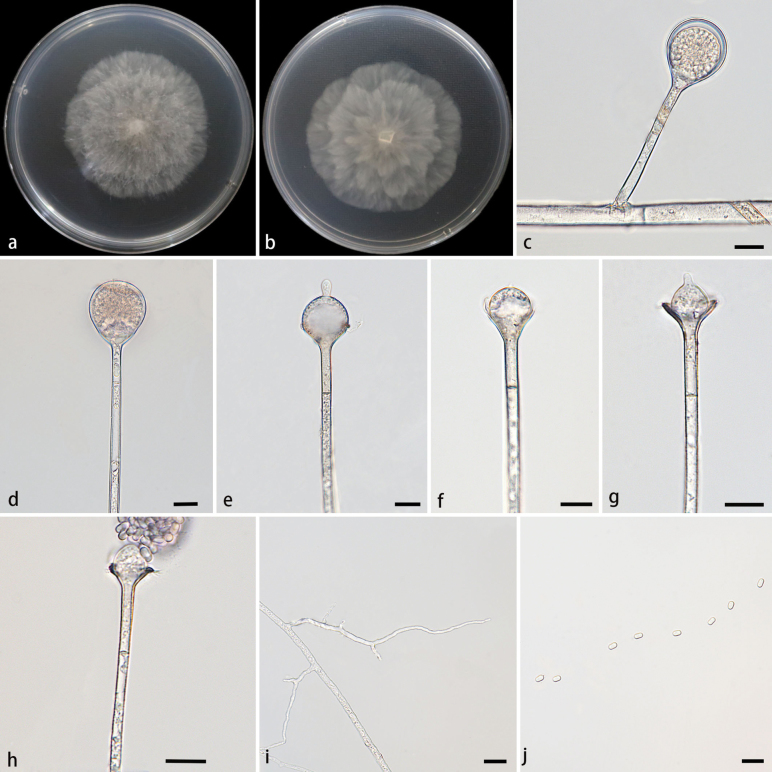
Morphologies of *Absidiaarrhiza* ex-holotype CGMCC 3.28540. **a, b.** Colonies on PDA; **a.** Obverse; **b.** Reverse; **c, d.** Sporangia; **e–h.** Columellae, collars, projections, and septa; **i.** Rhizoids; **j.** Sporangiospores. Scale bars: 10 μm (**c–j**).

##### Etymology.

The epithet *arrhiza* (Lat.) refers to producing few rhizoids in this species.

##### Description.

Hyphae branched, hyaline at first, gradually becoming brown when mature, aseptate when young, septate with age. Stolons branched, smooth, hyaline, brownish, septate, 3.7–9.7 µm in diameter. Rhizoids root-like, hyaline, poorly developed, simply branched. Sporangiophores arising from stolons, erect or slightly bent, single or 2–3 in whorls, unbranched, hyaline, 10.6–150.5 µm long, 2.4–5.3 µm wide, with a septum 14.4–18.7 µm below apophyses. Sporangia globose, multi-spored, smooth, deliquescent-walled, colorless when young, brownish when old, 10.5–38.8 µm long, 10.6–35.8 µm wide. Columellae subglobose to globose, smooth, hyaline, 5.0–11.5 µm long, 7.5–17.0 µm wide. Apophyses distinct, funnel-shaped, hyaline, slightly pigmented, 4.0–8.8 μm high, 2.1–4.5 µm wide at the base, and 5.0–17.6 µm wide at the top. Projections mostly pacifier-like, 0.7–6.5 µm long, 1.5–3.6 µm wide. Collars present or absent; if present, 1.8–4.9 µm long. Sporangiospores cylindrical, slightly concave in the center, smooth, hyaline, 2.8–4.5 µm long, 1.4–2.7 µm wide. Chlamydospores absent. Zygospores not found.

##### Culture characteristics.

Colonies on PDA in darkness at 25 °C for 7 days, growing slowly, reaching 79 mm in diameter, indicating an average growth rate of approximately 10.3–11.2 mm/d, hyaline at first, gradually becoming brown, irregular concentric ring zonate, petaloid, irregular at reverse.

##### Maximum growth temperature.

33 °C.

##### Additional specimen examined.

China • Yunnan Province, Xishuangbanna Dai Autonomous Prefecture, Menghai County G219 (Xijing Line) (22°11'02"N, 100°17'22"E, altitude 1492.96 m), from a soil sample, 7 July 2024, Z.Y. Ding and X.Y. Liu, living culture XG09770-6.

##### Notes.

According to the SSU-ITS-LSU-*Act*-*TEF1α* sequences, two strains of the *Absidiaarrhiza* sp. nov. were formed into an independent branch with full support (MLBV = 100, BIPP = 1.00; Fig. 1), closely related to *A.chinensis* (Zhao et al. 2023). These two species were obviously different in the morphology of sporangiophores, sporangia, apophyses, and sporangiospores. The maximum length of the sporangiophores of *A.arrhiza* was shorter than those of *A.chinensis* (150.5 µm vs. 220.0 µm). *A.arrhiza* mainly owned the shape of globose sporangia, while *A.chinensis* had two shapes, including globose and pyriform. The apophyses of *A.arrhiza* were higher than those of *A.chinensis* (4.0–8.8 μm vs. 3.5–7.0 µm). The sporangiospores of *A.arrhiza* were mainly cylindrical, while those of *A.chinensis* were mainly cylindrical to oval. *Z*ygospores were absent in *A.arrhiza*, while present in *A.chinensis*. Physiologically, the maximum growth temperature of *A.arrhiza* was higher than that of *A.chinensis* (33 °C vs. 30 °C) (Zhao et al. 2023).

#### ﻿ Absidia simplex

Taxon classificationFungiMucorales

Z.Y. Ding, Yang Jiang, Yi Xin Wang & X.Y. Liu sp. nov.

D84EC28E-3286-5883-95F7-4C338F37FA0D

Fungal Names: FN 572260

Fig. 3

##### Type.

China • Yunnan Province, Longling County, Mengnuo Town (24°31'25"N, 99°00'42"E, altitude 1319.65 m), from a soil sample, 8 July 2024, Z.Y. Ding and X.Y. Liu, holotype HMAS 353364, ex-holotype living culture CGMCC 3.28541 (= XG10012-9).

**Figure 3.**
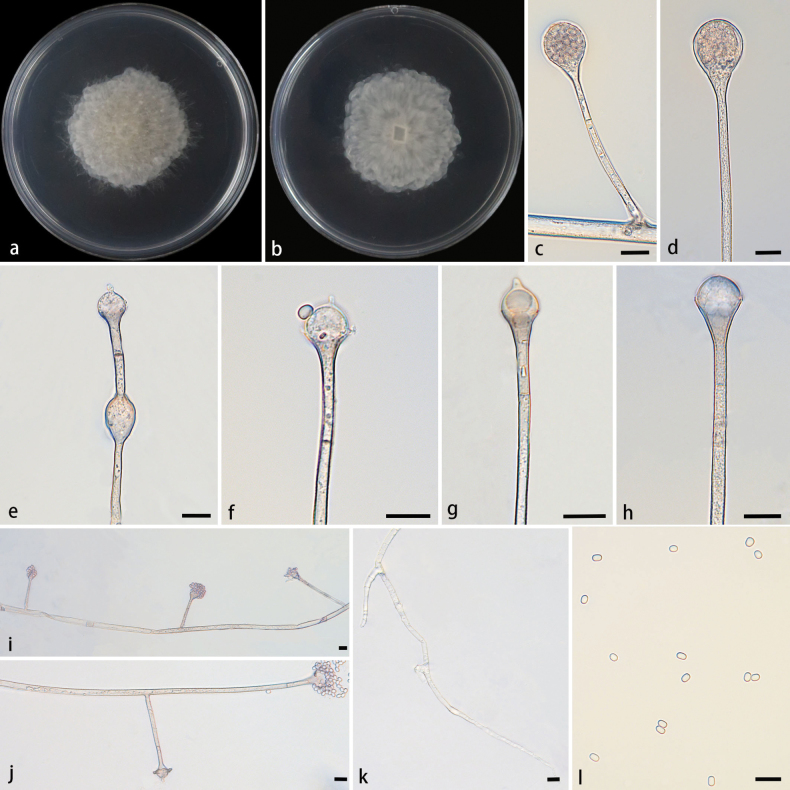
Morphologies of *Absidiasimplex* ex-holotype CGMCC 3.28541. **a, b.** Colonies on PDA; **a.** Obverse; **b.** Reverse; **c, d.** Sporangium; **e–h.** Columellae, collars, projections, and septa; **i.** Monopodial sporangiophores; **j.** Branched sporangiophores; **k.** Rhizoids; **l.** Sporangiospores. Scale bars: 10 μm (**c–l**).

##### Etymology.

The epithet *simplex* (Lat.) refers to the simple branching pattern of sporangiophores.

##### Description.

Hyphae branched, hyaline when young, light brownish when old, aseptate initially, septate with age. Stolons branched, smooth, hyaline, brownish, septate, 2.5–10.0 µm in diameter. Rhizoids finger-like, hyaline, poorly developed, mostly unbranched, occasionally simply branched. Sporangiophores arising from stolons, erect or slightly bent, commonly monopodial, unbranched or branched 1–2 times, never in whorls, hyaline, 6.2–359.2 µm long, 2.3–4.9 µm wide, with a septum 10.3–16.1 µm below apophyses, occasionally with a swelling below a septum. Sporangia globose to pyriform, multi-spored, subhyaline, smooth, deliquescent-walled, colorless when juvenile, dusky brown when old, 8.5–31.6 µm long, 8.8–26.1 µm wide. Columellae globose, subglobose, conical, smooth, subhyaline or hyaline, 2.1–10.2 µm long, 5.6–14.7 µm wide. Apophyses obvious, funnel-shaped, subhyaline or hyaline, slightly pigmented, 2.8–6.8 μm high, 1.7–3.4 µm wide at the base, and 5.0–14.8 µm wide at the top. Projections mostly cylindrical, always present, rarely absent, subhyaline or hyaline, 1.0–2.8 µm long, 0.8–2.2 µm wide. Collars present or absent; if present, 1.3–4.8 µm long. Sporangiospores cylindrical, oval, slightly concave in the center, smooth, hyaline, 2.2–6.2 µm long, 1.7–3.6 µm wide. Chlamydospores absent. Zygospores not found.

##### Culture characteristics.

Colonies on PDA at 25 °C for 7 days, reaching 60 mm in diameter, indicating an average growth rate of approximately 7.8–8.6 mm/d, hyaline initially, light brownish when old, irregularly concentrically zonate with ring, sporadically petalous at margin, irregular in reverse.

##### Maximum growth temperature.

29 °C.

##### Additional specimen examined.

China • Yunnan Province, Longling County, Mengnuo Town (24°31'25"N, 99°00'42"E, altitude 1319.65 m), from a soil sample, 8 July 2024, Z.Y. Ding and X.Y. Liu, living culture XG10012-8.

##### Notes.

Based on the SSU-ITS-LSU-*Act*-*TEF1α* sequences, two strains of the *A.simplex* sp. nov. formed into an independent branch with full support (MLBV = 100, BIPP = 1.00; Fig. 1), closely related to *A.panacisoli* (Zhang et al. 2018). These two species evidently differed in the morphology of stolons, sporangiophores, columellae, and sporangiospores. The maximum width of the stolons of *A.simplex* was narrower than those of *A.panacisoli* (10.0 µm vs. 10.5 µm). The maximum sporangiophore length of *A.simplex* was wider than that of *A.panacisoli* (4.9 µm vs. 2.8 µm). The columellae of *A.simplex* presented conical shapes, while *A.panacisoli* were absent. The sporangiospores of *A.simplex* possessed two shapes: cylindrical and oval, while those of *A.panacisoli* were mainly short and cylindrical. The zygospores of *A.simplex* were absent, while those of *A.panacisoli* were evident. Physiologically, the maximum growth temperature of *A.simplex* was lower than that of *A.panacisoli* (29 °C vs. 32 °C) (Zhang et al. 2018).

#### ﻿ Absidia viridis

Taxon classificationFungiMucorales

Z.Y. Ding, Yang Jiang, Yi Xin Wang & X.Y. Liu sp. nov.

D05EA14A-CB3F-5BB7-A6BC-F3EB95BC16FA

Fungal Names: FN 572261

Fig. 4

##### Type.

China • Yunnan Province, Mojiang Hani Autonomous County, Puer City (23°29'93"N, 101°39'17"E, altitude 1471.21 m), from soil, 4 July 2024, Z.Y. Ding and X.Y. Liu, holotype HMAS 353365, ex-holotype living culture CGMCC 3.28539 (= XG09563-3).

**Figure 4.**
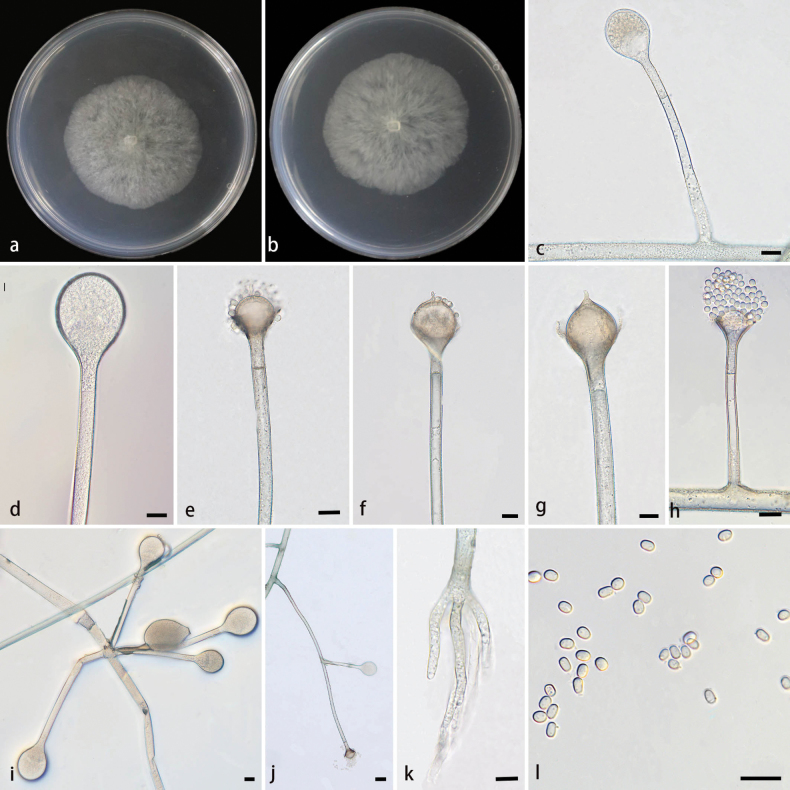
Morphologies of *Absidiaviridis* ex-holotype CGMCC 3.28539. **a, b.** Colonies on PDA; **a.** Obverse; **b.** Reverse; **c, d.** Sporangium; **e–h.** Columellae, collars, projections, and septa; **i.** Whorled sporangium; **j.** Branched sporangiophores; **k.** Rhizoids; **l.** Sporangiospores. Scale bars: 10 μm (**c–l**).

##### Etymology.

The *viridis* (Lat.) refers to the light green colony on PDA.

##### Description.

Hyphae branched, hyaline at first, light green when mature, aseptate when young, septate with age. Stolons branched, smooth, hyaline, brownish, septate, 7.8–12.1 µm in diameter. Rhizoids finger-like, rarely present, hyaline, unbranched. Sporangiophores arising from stolons, erect or slightly bent, single or 2–4 in whorls, unbranched or branched 1–2 times, hyaline, 9.7–472.5 µm long, 6.5–10.7 µm wide, with a septum 10.4–18.3 µm below apophyses. Sporangia circular, elliptic, multi-spored, smooth, deliquescent-walled, colorless in youth, pigmented when old, 21.0–59.6 µm long, 17.7–51.4 µm wide. Columellae mostly spherical, occasionally hemisphere, conical, smooth, hyaline, 8.7–28.4 µm long, 11.1–31.7 µm wide. Apophyses funnel-shaped, hyaline, slightly pigmented, 5.4–14.3 μm high, 6.8–18.3 µm wide at the base, and 12.2–26.5 µm wide at the top. Projections evident, mostly strip-shaped, 2.2–7.8 µm long, 1.6–5.9 µm wide. Collars present or absent; if present, 5.4–10.2 µm long. Sporangiospores cylindrical, smooth, hyaline, 2.1–4.2 µm long, 1.6–3.1 µm wide. Chlamydospores absent. Zygospores not found.

##### Culture characteristics.

Colonies on PDA at 25 °C for 7 days, reaching 80 mm in diameter, exhibiting an average growth rate of approximately 9.0–11.4 mm/day, hyaline at first, light green when mature, regular in reverse.

##### Maximum growth temperature.

29 °C.

##### Additional specimen examined.

China • Yunnan Province, Mojiang Hani Autonomous County (23°29'93"N, 101°39'17"E, altitude 1471.21 m), Puer City, from a soil sample, 4 July 2024, Z.Y. Ding and X.Y. Liu, living culture XG09563-2.

##### Notes.

In the phylogenetic tree of SSU-ITS-LSU-*Act*-*TEF1α*, two strains of the *A.viridis* sp. nov. formed a fully supported independent clade (MLBV = 100, BIPP = 1.00; Fig. 1), closely related to *A.varians* (Zhao et al. 2023). These two species were sensibly distinguished by the morphology of stolons, rhizoids, sporangiophores, columellae, apophyses, projections, sporangiospores. The maximum width of the stolons of *A.viridis* were wider than those of *A.varians* (12.1 µm vs. 8.5 µm). *A.viridis* was significantly different from *A.varians* in terms of rhizoid: the former was finger-like and unbranched, while the latter was root-like and multi-branched. The maximum sporangiophores length of *A.viridis* were shorter than those of *A.varians* (472.5 µm vs. 480.0 µm). The columellae of *A.viridis* presented conical shape, but *A.varians* absented. A single projection of one shape was observed at the apex of columellae in *A.viridis*, while 1–2 projections of three shapes in *A.varians*. The apophyses of the top width and base width in *A.viridis* were wider than those in *A.varians*, both at the top (12.2–26.5 µm vs. 10.5–22.5 µm) and at the base (6.8–18.3 µm vs. 5.5–11.0 μm). The apophysis-underneath septum grew at a closer distance in *A.viridis* than in *A.varians* (10.4–18.3 µm vs. 13.5–19.0 µm). The sporangiospores shape of *A.viridis* possessed mainly one kind, while *A.varians* possessed three kinds. Physiologically, the maximum growth temperature of *A.viridis* was one degree higher than that of *A.varians* (29 °C vs. 28 °C) (Zhao et al. 2023).

#### ﻿ Absidia sphaerica

Taxon classificationFungiMucorales

Z.Y. Ding, Yang Jiang, Yi Xin Wang & X.Y. Liu sp. nov.

1B24DFA6-D8DC-5D62-BB7D-D43D5AC13598

Fungal Names: FN 572262

Fig. 5

##### Type.

America • from a soil sample, X.Y. Liu, holotype HMAS 353366, ex-holotype living culture CGMCC 3.28542 (= XY00690).

**Figure 5.**
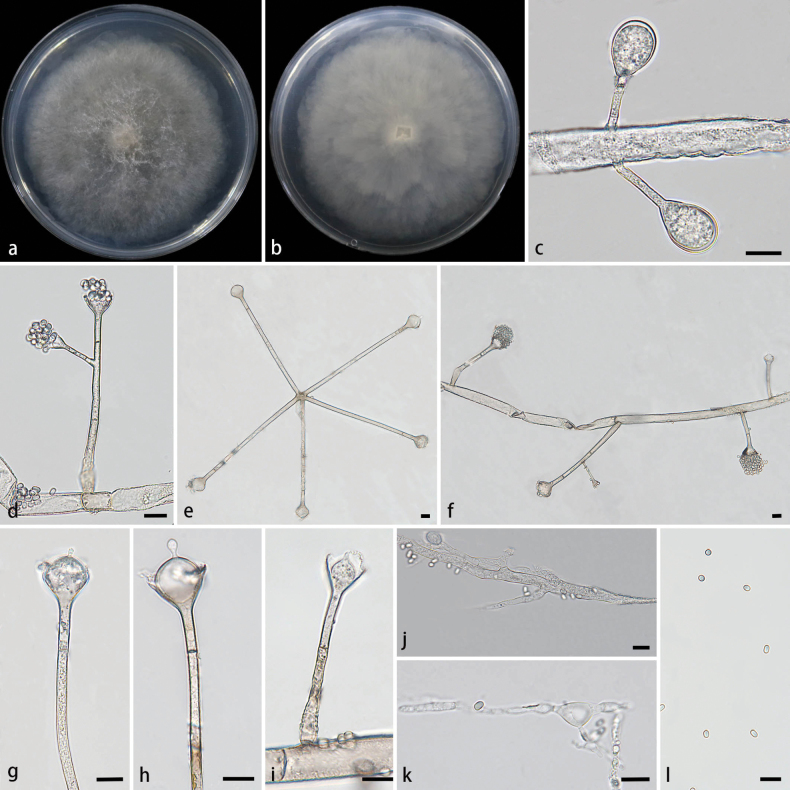
Morphologies of *Absidiasphaerica* ex-holotype CGMCC 3.28542. **a, b.** Colonies on PDA; **a.** obverse; **b.** Reverse; **c.** Sporangium; **d.** Branched sporangiophores; **e.** Whorled sporangium; **f.** Monopodial sporangiophores; **g–h.** Columellae, collars, projections, and septa; **j.** Rhizoids with a swelling; **k.** Zygospores; **l.** Sporangiospores. Scale bars: 10 μm (**c–l**).

##### Etymology.

The *sphaerica* (Lat.) refers to the spherical columellae.

##### Description.

Hyphae branched, hyaline at first, light brown when mature, aseptate when young, septate with age. Stolons branched, smooth, hyaline, septate, 6.3–10.1 µm in diameter. Rhizoids root-like, hyaline, rarely branched, occasionally with a swelling. Sporangiophores beginning with stolons, erect or slightly bent, single or 2–5 in whorls, unbranched or branched 1–2 times, hyaline, 11.7–171.0 µm long, 2.7–4.6 µm wide, with one to two septa 6.4–18.2 µm below apophyses. Sporangia spherical, pear-shaped, multi-spored, smooth, deliquescent-walled, colorless in youth, pigmented when old, 6.9–37.0 µm long, 6.6–30.9 µm wide. Columellae globose, subglobose, smooth, hyaline, 1.2–20.2 µm long, 3.5–20.9 µm wide. Apophyses funnel-shaped, hyaline, slightly pigmented, 5.4–14.3 μm high, 1.3–5.0 µm wide at the base, and 3.3–16.3 µm wide at the top. Projections evident, strip-shaped, papillary, or pacifier-like, 2.0–7.3 µm long, 1.5–3.6 µm wide. Collars present or absent; if present, 1.4–6.4 µm long. Sporangiospores mostly cylindrical, occasionally globose, smooth, hyaline, 2.7–3.6 µm long, 1.2–2.2 µm wide. Chlamydospores absent. Zygospores not found.

##### Culture characteristics.

Colonies on PDA at 25 °C for 7 days, reaching 79 mm in diameter, exhibiting an average growth rate of approximately 10.2–11.3 mm/d, hyaline at first, light brown when mature, regular in reverse.

##### Maximum growth temperature.

33 °C.

##### Additional specimen examined.

The United States of America • from a soil sample, latitude, longitude, and altitude unknown, X.Y. Liu, living culture XY00690-1.

##### Notes.

Based on the SSU-ITS-LSU-*Act*-*TEF1α* phylogenetic tree, two strains of the *A.sphaerica* sp. nov. formed a fully supported independent lineage (MLBV = 100, BIPP = 1.00; Fig. 1), closely related to *A.medulla* (Zong et al. 2021a). Although similar, they were different in terms of morphological features such as rhizoids, stolons, sporangiophores, sporangia, columellae, apophyses, projections, and sporangiospores. *A.sphaerica* in rhizoids had no spine-like structures, while *A.medulla* presented them. The maximum width of the stolons of *A.sphaerica* was wider than those of *A.medulla* (10.1 µm vs. 6.5 µm). The maximum sporangiophore length of the former was much shorter than that of the latter (171.0 µm vs. 220 µm). In addition, *A.sphaerica* was smaller than *A.medulla* in sporangia (6.9–37.0 × 6.6–30.9 μm vs. 12–41 × 11.5–32.5 μm). The columellae in *A.sphaerica* were slightly shorter than in *A.medulla* (1.2–20.2 µm vs. 8.5–20.5 µm), accompanied by a longer projection at its top (2.0–7.3 µm vs. 1–4.5 µm). The apophyses of *A.sphaerica* were not only shorter in height than *A.medulla* (1.2–7.5 µm vs. 3.0–8.5 µm) but also narrower in top width (3.3–16.31 µm vs. 7.5–17.5 µm). In addition, the septum was closer to apophyses in *A.sphaerica* than in *A.medulla* (6.4–18.2 µm vs. 12.5–27.5 µm). The sporangiospores in *A.sphaerica* are smaller than in *A.medulla* (2.7–3.6 µm × 1.2–2.2 µm vs. 3.0–4.5 µm × 2.0–3.5 µm). Physiologically, the maximum growth temperature of *A.sphaerica* was lower than that of *A.medulla* (33 °C vs. 32 °C) (Zong et al. 2021a).

## ﻿Discussion

The genus *Absidia* is mainly found in soil habitats in tropical, subtropical, and temperate climates (Zhang et al. 2018; Tao et al. 2024). Yunnan Province has a temperate to tropical monsoon climate. Based on molecular, morphological, and physiological data, three new species of *Absidia* were isolated from Yunnan soils. A fourth species originated from soil in the United States of America, but with unclear geographical information.

Phylogenetic analyses provided strong and robust evidence for fungal classification. The nucleotide sequence of ITS has proved to be a universal barcode within the genus *Absidia* (Hoffmann et al. 2007, 2013; Walther et al. 2013; Ariyawansa et al. 2015). However, identification of *Absidia* species solely by ITS sequences can sometimes yield unreliable results (Zhang et al. 2018). Therefore, LSU, SSU, or other nucleotide sequences are recommended to be combined (Hoffmann et al. 2007; Walther et al. 2013).

In this study, phylogenetic analyses of eight strains using combined SSU-ITS-LSU-Act-TEF1α sequences revealed four robust monophyletic clades (Fig. 1). *Absidiaarrhiza* was closely related to *A.chinensis* (MLBV = 100, BIPP = 1.00), *A.simplex* was closely related to *A.panacisoli* (MLBV = 100, BIPP = 1.00), *A.viridis* was associated with *A.varians* (MLBV = 100, BIPP = 1.00), and *A.sphaerica* was a sister clade to *A.medulla* (MLBV = 100, BIPP = 1.00). Compared to *A.chinensis*, the new species *A.arrhiza* has shorter sporangiophores, lower branching frequency, smaller sporangia, larger apophyses, and no zygospores. Compared with *A.panacisoli*, *A.simplex* has longer sporangiophores and larger sporangia. Additionally, swelling was observed in the sporangiophores of *A.simplex* but not in *A.panacisoli*. Importantly, *A.simplex* produces no zygospores, whereas *A.panacisoli* does. Finally, the apophyses of *A.simplex* are shorter than those of *A.panacisoli*. The sporangiophores of *A.viridis* are shorter than those of *A.varians*. The sporangiospores and projections of *A.viridis* have mainly one shape, while *A.varians* has three. Moreover, the columellae of *A.viridis* are smaller than those of *A.varians*. There is also a significant difference in rhizoid formation between *A.viridis* and *A.varians*, with *A.viridis* having digital-like rhizoids and *A.varians* having root-like rhizoids. The new species *A.sphaerica* has shorter sporangiophores and fewer whorls than *A.medulla*. Sporangia, sporangiospores, and apophyses of *A.sphaerica* are all smaller than those of *A.medulla*, whereas the projections of *A.sphaerica* are longer than those of *A.medulla*.

Hoffmann et al. (2007) comprehensively studied the molecular data, morphology, and physiology of *Absidia* s.l. and divided it into three categories: thermotolerant *Lichtheimia* (maximum growth temperature, MGT ≥ 37 °C), mesophilic *Absidia* s.s. (MGT 25–34 °C), and mycoparasitic *Lentamyces* (MGT ≤ 30 °C). Recent studies support that the maximum growth temperature of *Absidia* strains does not reach 37 °C (Zhao et al. 2022b, 2023). This study further confirms this view: the maximum growth temperatures of *A.arrhiza*, *A.simplex*, *A.viridis*, and *A.sphaerica* were 33 °C, 29 °C, 29 °C, and 33 °C, respectively.

Scientists have been paying increasing attention to the diversity and ecological distribution of fungi, providing a reliable foundation for an in-depth understanding of the Fungi kingdom (Zhao et al. 2021; Zong et al. 2021a; Liu et al. 2021; Cai et al. 2022). It has been found that most *Absidia* species were isolated from moist and dark soil environments in tropical, subtropical, and temperate regions and less frequently from feces, fallen leaves, and other habitats (Zhang et al. 2018; Cordeiro et al. 2020; Lima et al. 2020; Zhao et al. 2021; Tao et al. 2024). The three new species reported here were discovered in moist soil in Yunnan Province, enriching the diversity of *Absidia* in temperate to subtropical areas and providing an effective reference for future exploration of *Absidia* biological resources.

## Supplementary Material

XML Treatment for Absidia arrhiza

XML Treatment for Absidia simplex

XML Treatment for Absidia viridis

XML Treatment for Absidia sphaerica

## ﻿Additional information

### Author ORCIDs

Zi-Ying Ding https://orcid.org/0009-0003-1618-5740

Xin-Yu Ji https://orcid.org/0009-0000-4121-9103

Meng-Fei Tao https://orcid.org/0009-0008-9053-1310

Yang Jiang https://orcid.org/0009-0001-5231-914X

Yi-Xin Wang https://orcid.org/0009-0001-5231-914X

Xiao-Yong Liu https://orcid.org/0000-0002-8808-010X

### Data availability

All of the data that support the findings of this study are available in the main text or Supplementary Information. The sequences were deposited in the GenBank database.

## Supplementary materials

Supplementary material 1 Supplementary data Data type docx Explanation note **table S1.** GenBank accession numbers of sequences used in this study; **figure S1.** The maximum likelihood phylogenetic tree of *Absidia* based on sequences of ITS rDNA. https://binary.pensoft.net/file/1360749This dataset is made available under the Open Database License (http://opendatacommons.org/licenses/odbl/1.0/). The Open Database License (ODbL) is a license agreement intended to allow users to freely share, modify, and use this Dataset while maintaining this same freedom for others, provided that the original source and author(s) are credited.Zi-Ying Ding, Xin-Yu Ji, Meng-Fei Tao, Yang Jiang, Wen-Xiu Liu, Yi-Xin Wang, Zhe Meng, Xiao-Yong Liu
