# Supplementary material for: ﻿Unveiling species diversity within early-diverging fungi from China IV: Four new species of Absidia (Cunninghamellaceae, Mucoromycota)
Source: MycoKeys. 2025 Jun 25;119:29–46. doi: 10.3897/mycokeys.119.147816 (PMC12238976; doi:10.3897/mycokeys.119.147816)
Supplement: Supplementary material 1 — Supplementary data [file mycokeys-119-029-s001.docx]

**Table S1.** GenBank accession numbers of sequences used in this study.

| Species | Strains | GenBank accession numbers | | | | |
| --- | --- | --- | --- | --- | --- | --- |
|  |  | SSU | ITS | LSU | Act | TEF–1α |
| *Absidia abundans* | XY09265 | NA | ON074697 | ON074681 | NA | NA |
| *A. abundans* | CGMCC 3.16255* | NA | NR_182590 | ON074683 | NA | NA |
| *A. abundans* | XY09274 | NA | ON074696 | ON074682 | NA | NA |
| *A. aguabelensis* | URM 8213* | NA | NR_189383 | NG_241934 | NA | NA |
| *A. alpina* | CGMCC 3.16104 | NA | OL678133 | NA | NA | NA |
| *A. ampullacea* | CGMCC 3.16054 | NA | MZ354138 | MZ350132 | NA | NA |
| *A. anomala* | CBS 125.68* | NA | MH859085 | MH870799 | NA | NA |
| *A. anomala* | FSU5798 | NA | EF030523 | NA | EF030535 | NA |
| ***A. arrhiza*** | **XG09770-6** | **PQ600262** | **PQ600859** | **PQ600253** | **PQ753532** | **PQ680678** |
| ***A. arrhiza*** | **CGMCC 3.28540*** | **PQ600263** | **PQ600860** | **PQ600254** | **PQ753533** | **PQ680679** |
| *A. biappendiculata* | CBS 187.64 | NA | MZ354153 | MZ350147 | MZ357438 | MZ357420 |
| *A. bonitoensis* | URM 7889* | NA | MN977786 | MN977805 | NA | NA |
| *A. brunnea* | CGMCC 3.16055* | NA | MZ354139 | MZ350133 | MZ357421 | MZ357403 |
| *A. caatinguensis* | URM 7156* | NA | NR_154704 | NG_058582 | NA | NA |
| *A. caerulea* | XY00608 | NA | OL620081 | NA | NA | NA |
| *A. caerulea* | XY00729 | NA | OL620082 | NA | NA | NA |
| *A. caerulea* | CBS101.36 | NA | MH855718 | MH867230 | NA | NA |
| *A. caerulea* | FSU767 | NA | AY944870 | NA | NA | NA |
| *A. californica* | CBS 314.78 | NA | JN205816 | MH872902 | NA | NA |
| *A. californica* | FSU4748 | EU736274 | AY944873 | EU736301 | EU736224 | EU736247 |
| *A. californica* | FSU4747 | EU736273 | AY944872 | EU736300 | AY944758 | EU736246 |
| *A. chinensis* | CGMCC 3.16057 | NA | MZ354141 | MZ350135 | MZ357422 | NA |
| *A. chinensis* | CGMCC 3.16056* | NA | MZ354140 | MZ350134 | NA | NA |
| *A. cinerea* | CGMCC 3.16062 | NA | MZ354146 | MZ350140 | MZ357427 | MZ357407 |
| *A. cornuta* | URM 6100* | NA | NR_172976 | MN625255 | NA | NA |
| *A. cuneospora* | CBS 101.59* | NA | MH857828 | MH869361 | NA | NA |
| *A. cuneospora* | FSU5890 | NA | EF030524 | NA | EF030533 | NA |
| *A. cylindrospora* | CBS 100.08 | NA | JN205822 | JN206588 | NA | NA |
| *A. digitula* | CGMCC 3.16058* | NA | MZ354142 | MZ350136 | MZ357423 | MZ357404 |
| *A. edaphica* | MFLU–20–0416* | MT394048 | MT396372 | MT393987 | MT410739 | NA |
| *A. edaphica* | MFLUCC 20–0088 | NG_074951 | NR_172305 | NG_075367 | MT410739 | NA |
| *A. frigida* | CGMCC 3.16201* | NA | NR_182565 | OM030223 | NA | NA |
| *A. fusca* | CBS 102.35* | NA | NR_103625 | NG_058552 | NA | NA |
| *A. gemella* | CGMCC 3.16202* | NA | OM108488 | OM030224 | NA | NA |
| *A. glauca* | CBS 129233 | NA | MH865253 | MH876693 | NA | NA |
| *A. glauca* | CBS 101.08* | NA | MH854573 | MH866105 | NA | NA |
| *A. glauca* | FSU660 | EU736275 | AY944879 | EU736302 | EU736225 | EU736248 |
| *A. globospora* | CGMCC 3.16031* | NA | NR_189829 | MW671544 | MZ357431 | MZ357412 |
| *A. globospora* | CGMCC 3.16035 | NA | MW671538 | MW671545 | MZ357432 | MZ357413 |
| *A. globospora* | CGMCC 3.16036 | NA | MW671539 | MW671546 | MZ357433 | MZ357414 |
| *A. heterospora* | CBS 101.29* | NA | JN206595 | MH866483 | NA | NA |
| *A. heterospora* | SHTH021 | JQ004928 | JN942683 | JN982936 | NA | NA |
| *A. jiangxiensis* | CGMCC 3.16105* | PP779719 | OL678134 | PP780377 | PP790577 | PP790569 |
| *A. jindoensis* | CNUFC–PTI1–1 | MF926626 | MF926622 | MF926616 | MF926510 | MF926513 |
| *A. koreana* | EML–IFS45–1* | KT321298 | KR030062 | KR030056 | KR030058 | KR030060 |
| *A. koreana* | XY00816 | NA | OL620083 | ON123771 | NA | NA |
| *A. koreana* | XY00596 | NA | OL620084 | NA | NA | NA |
| *A. lobata* | CGMCC 3.16256 | NA | ON074690 | ON074679 | NA | NA |
| *A. longissima* | CGMCC 3.16203* | NA | NR_182566 | OM030225 | NA | NA |
| *A. macrospora* | CBS 697.68* | NA | HM849704 | NA | NA | NA |
| *A. macrospora* | FSU4746 | EU736276 | AY944882 | EU736303 | AY944760 | EU736249 |
| *A. medulla* | CGMCC 3.16034 | NA | NR_189832 | MW671549 | MZ357436 | MZ357417 |
| *A. montepascoalis* | URM 8218 | NA | NR_172995 | NA | NA | NA |
| *A. multispora* | URM 8210* | NA | MN953780 | MN953782 | NA | NA |
| *A. nigra* | CBS 127.68* | NA | NR_173068 | MZ350146 | MZ357437 | MZ357419 |
| *A. nigra* | CGMCC 3.16059 | NA | MZ354143 | MZ350137 | MZ357424 | MZ357405 |
| *A. nigra* | CGMCC 3.16060 | NA | MZ354144 | MZ350138 | MZ357425 | MZ357406 |
| *A. oblongispora* | CGMCC 3.16061 | NA | MZ354145 | MZ350139 | MZ357426 | NA |
| *A. ovalispora* | CGMCC 3.16019 | NA | NR_176748 | MW264131 | NA | NA |
| *A. panacisoli* | SYPF 7183* | MF522179 | MF522181 | MF522180 | NA | MF624251 |
| *A. pararepens* | XY00631 | NA | OL620085 | ON123774 | NA | NA |
| *A. pararepens* | XY00615 | NA | OL620086 | NA | NA | NA |
| *A. pararepens* | XY05899 | NA | OL620087 | NA | NA | NA |
| *A. pararepens* | CCF 6352 | NA | MT193669 | MT192308 | NA | NA |
| *A. pernambucoensis* | URM<BRA>7219 | NA | MN635568 | MN635569 | NA | NA |
| *A. pseudocylindrospora* | EML–FSDY6–2 | KU923819 | KU923817 | KU923814 | KU923815 | NA |
| *A. pseudocylindrospora* | CBS 100.62* | NA | NR_145276 | MH869688 | NA | NA |
| *A. psychrophilia* | FSU4745 | EU736279 | AY944874 | EU736306 | AY944762 | EU736252 |
| *A. purpurea* | CGMCC 3.16106 | NA | OL678135 | NA | NA | NA |
| *A. radiata* | CGMCC 3.16257 | NA | ON074698 | ON074684 | NA | NA |
| *A. radiata* | XY09330–1 | NA | ON074699 | ON074685 | NA | NA |
| *A. repens* | CBS 115583* | NA | NR_103624 | NG_058551 | NA | NA |
| ***A. simplex*** | **XG10012-8** | **PQ600264** | **PQ600861** | **PQ600255** | **PQ686229** | **PQ661464** |
| ***A. simplex*** | **CGMCC 3.28541*** | **PQ600265** | **PQ600862** | **PQ600256** | **PQ686230** | **PQ661465** |
| ***A. sphaerica*** | **XY00690-1** | **PQ600268** | **PQ600865** | **PQ600259** | **PQ777148** | **PQ677802** |
| ***A. sphaerica*** | **CGMCC 3.28542*** | **PQ600269** | **PQ600866** | **PQ600260** | **PQ777149** | **PQ677803** |
| *A. saloaensis* | URM 8209* | NA | MN953781 | MN953783 | NA | NA |
| *A. sichuanensis* | CGMCC 3.16258* | NA | NR_182589 | ON074688 | NA | NA |
| *A. soli* | MFLU–20–0414* | MT394049 | MT396373 | MT393988 | NA | NA |
| *A. spinosa* | FSU551 | EU736280 | AY944887 | EU736307 | EU736227 | EU736253 |
| *A. stercoraria* | EML–DG8–1* | NG_065640 | KU168828 | KT921998 | KT922000 | KT922002 |
| *A. sympodialis* | CGMCC 3.16063* | NA | MZ354147 | MZ350141 | NA | NA |
| *A. sympodialis* | CGMCC 3.16064 | NA | MZ354148 | MZ350142 | NA | MZ357408 |
| *A. terrestris* | FMR 14989* | NA | LT795003 | LT795005 | NA | NA |
| *A. turgida* | CGMCC 3.16032* | NA | NR_189830 | NG_241931 | MZ357434 | MZ357415 |
| ***A. viridis*** | **XG09563-2** | **PQ600266** | **PQ600863** | **PQ600257** | **PQ753530** | **PQ661233** |
| ***A. viridis*** | **CGMCC 3.28539*** | **PQ600267** | **PQ600864** | **PQ600258** | **PQ753531** | **PQ661234** |
| *A. varians* | CGMCC 3.16065* | NA | MZ354149 | MZ350143 | MZ357428 | MZ357409 |
| *A. virescens* | CGMCC 3.16066* | NA | MZ354150 | MZ350144 | MZ357429 | MZ357410 |
| *A. virescens* | CGMCC 3.16067 | NA | MZ354151 | MZ350145 | MZ357430 | MZ357411 |
| *A. xinjiangensis* | CGMCC 3.16107* | NA | OL678136 | NA | NA | NA |
| *A. yunnanensis* | XY09528 | NA | ON074701 | ON074686 | NA | NA |
| *A. yunnanensis* | CGMCC 3.16259* | NA | NR_182591 | NG_149054 | NA | NA |
| *A. zonata* | CGMCC 3.16033* | NA | NR_189831 | MW671548 | MZ357435 | MZ357416 |
| *A. zygospora* | MFLUCC 23#0061* | NA | OR104965 | OR104992 | NA | NA |
| *A. zygospora* | RSPG 214 | NA | KC478527 | NA | NA | NA |
| *A. zygospora* | ANG28 | NA | DQ914420 | NA | NA | NA |
| *Cunninghamella blakesleeana* | CBS 133.27* | NA | JN205865 | MH866397 | NA | KJ156479 |
| *C.blakesleeana* | CBS 782.68 | NA | JN205869 | MH870950 | NA | NA |
| *C. elegans* | CBS 160.28* | NA | MH854961 | NA | NA | KJ156470 |
| *C. elegans* | CBS 167.53 | NA | MH857146 | HM849700 | NA | NA |

Notes: New species proposed herein are shown in bold. The Ex-type or ex-holotype strains are marked with an asterisk "*". The "NA" stands for "unavailable".


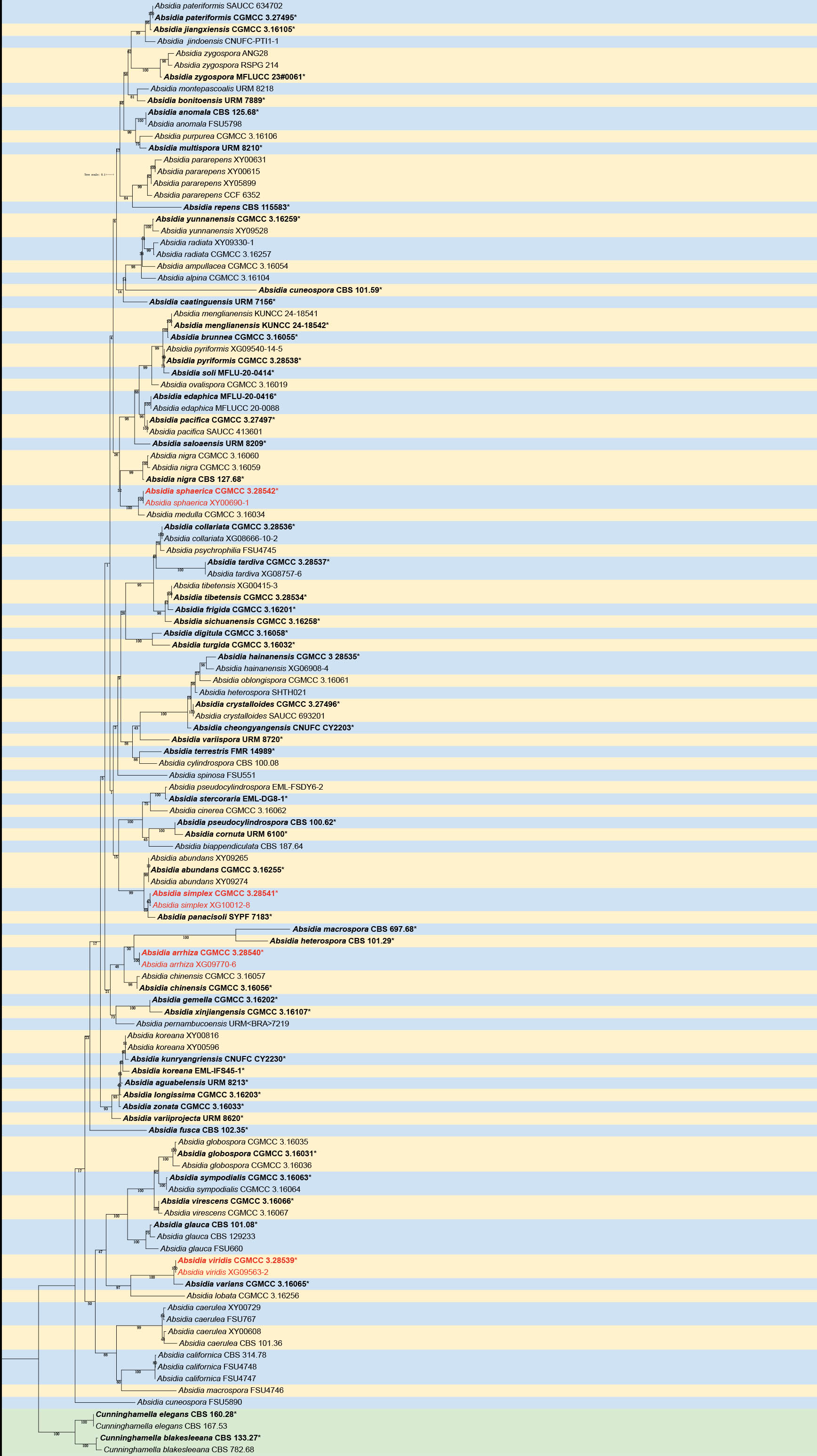


**Figure S1.** The maximum likelihood phylogenetic tree of *Absidia* based on sequences of ITS rDNA.
